# 8.C. Workshop: Networking for life: How European regions develop/strengthen cross-border health

**DOI:** 10.1093/eurpub/ckac129.484

**Published:** 2022-10-25

**Authors:** Organised by: Healthacross Initiative, Health Agency of Lower Austria (Austria), Chair persons: Julia Winkler (Austria), Bettina Menne (Denmark)

## Abstract

The WHO European Programme of Work (2020-2025) emphasizes the importance of “supporting local living environments that enable health and well-being”. Through engaging with regulatory arrangements that support an environment that responds to citizens’ concerns for safer, healthier and better living, the EPW intertwines with the aims of the EPH Conference to promote population health and to strengthen health systems. The Covid-19 pandemic shows that health threats do not stop at national borders. Different responses amongst cross-border regions, based on national policies in terms of Public Health and Social Measures (PHSM), may even weaken their effectiveness. The importance of cooperation across border is not only relevant in the framework of pandemic preparedness and responses, but in many other fields: healthcare cooperation, emergency medical care, medico-social cooperation and health promotion. All have shown to be beneficial to population health when developed at the subnational level across border regions. Consequently, cross-border health (care) gained importance in recent years and there are various border regions who showcase successful cross-border cooperation in the field of health. Projects are being implemented along neighbouring European regions, translating into improved access to healthcare for the border populations, promoting prevention and health education as well as increasing healthcare availability and equity. Via various health networks, border regions can learn to engage with neighbours and build up tailor-made health services for their citizens. More so, through active participation in various well-established public health networks, the exchange of ideas, knowledge and solutions to strengthen cross-border health becomes part of region's daily work. Through strong networks and partnerships, joint solutions for a strengthened citizens’ health in rural areas could be found. Creating synergies between the healthcare capacities of the two sides of the border, collaboration between border area medical teams, access to equipment located one or other side of the border can make a positive impact on the users of health services and facilities. In this round table workshop, the role and impact of health networks on a sub-national level (regional) will be further examined. Based on selected well-established health networks, their work and value will be outlined.

The Keynote speech will be given by the Coordinator of the WHO Europe Regions for Health Network, Dr. Bettina Menne. Subsequently, 3-4 well-established health networks will take the floor with a short presentation (5 min) in order to present their ‘business case’ and value. The presentations will be followed by a short round table discussion in order to highlight the role and strengths European health networks can bring to regions in order to improve cross-border healthcare (WHO RHN, Healthacross /Lower Austria, euPrevent, AEBR, EUREGHA).

**Key messages:**

• Role and impact of health networking for cross-border health on a sub-national level.

• Outlining the benefits and challenges for border regions to participate in health networks in order to strengthen cross-border health (also during Covid-19).

